# Perceived stress, family impact, and changes in physical and social daily life activities of children with chronic somatic conditions during the COVID-19 pandemic

**DOI:** 10.1186/s12889-022-13544-8

**Published:** 2022-06-03

**Authors:** Anne Krijger, Karolijn Dulfer, Hedy van Oers, Lorynn Teela, Brita de Jong-van Kempen, Anne van Els, Lotte Haverman, Koen Joosten

**Affiliations:** 1 Intensive Care Unit, Department of Pediatrics and Pediatric Surgery, Erasmus Medical Centre, Sophia Children’s Hospital, PO box 2060, 3000 CB Rotterdam, the Netherlands; 2 Amsterdam UMC, Location University of Amsterdam, Emma Children’s Hospital, Child and Adolescent Psychiatry & Psychosocial Care, Amsterdam Reproduction and Development, Amsterdam Public Health, Meibergdreef 9, Amsterdam, The Netherlands; 3 Department of Pediatrics, Medical Center Leeuwarden, Leeuwarden, the Netherlands; 4 Amsterdam UMC, Department of Pediatrics, Location University of Amsterdam, Meibergdreef 9, Amsterdam, the Netherlands

**Keywords:** COVID-19, Children, Chronic disease, Perceived stress, Social health, Physical activity, Financial situation

## Abstract

**Background:**

The COVID-19 pandemic has inevitably affected children and their families. This study examines the impact of the COVID-19 measures in children with chronic somatic conditions (CSC) and their parents and compares them with a Dutch general population sample.

**Methods:**

We included a sample of children with CSC (0–18 years, *n* = 326) and compared them with children (8–18 years, *n* = 1,287) from the Dutch general population. Perceived stress, coping, social interaction with friends and family, physical activity, eating behavior, family support, parenting perception, and financial situation were assessed once with the self-reported and parent-reported COVID-19 child check questionnaire, between November 2020 and May 2021. Comparisons between the two samples were made by using t-tests and chi square tests.

**Results:**

The proportion of children who reported being less physically active and having less social interaction with friends since the COVID-19 pandemic was higher in children with CSC than in children from the general population. Children with CSC and their parents experienced less stress than children and parents from the general population. Moreover, parents of children with CSC aged 0–7 years and parents of children aged 8–18 years from the general population experienced less support and more financial deterioration than parents of children with CSC aged 8–18 years. In the parents from the general population only, this deteriorated financial situation was associated with more stress, worse family interaction and parenting perception, and less received support.

**Conclusions:**

The impact of COVID-19 on children with CSC and their parents differed from those in the general population. Addressing the collateral damage of COVID-19 measures in children and their families may give direction to policy and potentially prevent lifelong impact.

**Supplementary Information:**

The online version contains supplementary material available at 10.1186/s12889-022-13544-8.

## Background

Early 2020, coronavirus disease 2019 (COVID-19) evolved from a local outbreak in Wuhan into a global pandemic. Despite children generally having milder forms of COVID-19, the COVID-19 pandemic likely had a significant impact on daily life of children and their families [1]. To prevent the spread of COVID-19 and the collapse of health care systems, imposed measures, such as social distancing and closure of schools and sports clubs, restricted the everyday life of children. These restrictions have presumably affected children’s behavior and well-being as well as their parents’ [2].

For children with chronic somatic conditions (CSC), defined as a diagnosis based on medical scientific knowledge, highly resistant to treatment, and lasting longer than three months [3], the impact of the COVID-19 measures might be different than in healthy children. Prior to COVID-19, children with CSC were already at higher risk of having impaired psychological wellbeing. Due to the often unexpected, uncontrollable, and functionally impairing nature of chronic conditions they are, for example, more vulnerable to experience stress [4]. In addition to the psychological impact, children with CSC may be faced with other disadvantages. Depending on the severity and degree of disability of their condition, children may be absent from school more often, for example due to frequent hospitalizations or outpatient visits. Regarding lifestyle, it was found that children with a somatic or psychiatric chronic disease had a poorer diet, engaged less in physical activity, spent more time watching television, and had less social interactions with friends than their healthy peers [5]. In the family context, the matter of a child with CSC may also have a detrimental impact. Parental stress is a common phenomenon and parental overprotection might hamper the development of the chronically ill child. The financial status might also be worse due to added caregiving demands and income loss [6]. A clear understanding of the impact of the COVID-19 pandemic in this vulnerable group may enable healthcare professionals to adequately support children with CSC and their parents.

Studies in children and adolescents from the general population have demonstrated that the COVID-19 pandemic had significant impact on psychological wellbeing, particularly resulting in more symptoms of stress, anxiety, and depression [2, 7–10]. Various factors may be underlying these psychological complaints, including disruption in school and physical activity routines, not being able to play outdoors, the lack of in-person contact with friends and extracurricular activities and boredom [8, 10]. Regarding daily activities, Dutch studies showed that children missed contact with their friends, were less physically active and spent more time using electronic screens during the COVID-19 pandemic than before [8, 11]. The psychological impact of COVID-19 in children with chronic conditions were found to be two-sided: i.e. leading to challenges as well as opportunities [12]. Challenges are heightened health anxiety, stress of disrupted routines and school closure, but also an increased risk of family stress and reduced access to support. Whereas opportunities can include increased time with family, reduced academic stress, the opportunity to build resilience, reduced access to substances, and more access to healthcare technology [12]. To date, few studies have compared the psychological impact of the COVID-19 pandemic in children with CSC and their parents to the impact in healthy children and their parents. In children with lung diseases -who are therefore more vulnerable to COVID-19- one study found more anxiety in children and parents than in healthy controls [13], whereas another study could only confirm this result in mothers, as they showed that healthy children experienced more anxiety [14]. Studies that compared children with CSC in general to healthy controls also found conflicting results [15–17]. Moreover, little is known about changes in daily life activities due to COVID-19 in children with CSC. To explore whether children with CSC and their families should be supported different than healthy controls, studies with larger sample sizes and a variety of chronic conditions are needed.

Therefore, the objectives of our study were 1) to compare the impact of the Dutch COVID-19 measures on perceived stress, coping, social interaction with friends, physical activity, and eating behavior in children aged 8–18 years with CSC and from the general population, 2) to assess the impact of the Dutch COVID-19 measures on perceived stress, family interaction, parenting perception, family support and financial situation in parents of children with CSC aged 0–18 years, 3) to compare the impact of the Dutch COVID-19 measures in parents of children with CSC aged 0–7 years to parents of children with CSC aged 8–18 years, and 4) to compare the impact of the Dutch COVID-19 measures on parents of children aged 8–18 years with CSC and from the general population.

## Methods

### The COVID-19 regulation timeline in the Netherlands

From October 14^th^ to December 14^th^ 2020, the second partial COVID-19 lockdown came into effect in the Netherlands [18]. In addition to the basic rules of hygiene, social distancing, wearing a face mask in public indoor spaces, working and staying at home as much as possible, all bars and restaurants were closed, shops had to close at 8 pm and it was allowed only to receive three guests at home. Starting December 14^th^ 2020, a hard lockdown was in effect, which included closure of schools, out-of-school care and daycare (except for socially vulnerable children and children with parents having an essential profession), non-essential shops and leisure facilities. Sports clubs were also closed, but children up to 17 years were allowed to play sports outside individually and in teams [18]. A curfew was effective from January 23^rd^ to April 28^th^ 2021. On February 8^th^ 2021, primary schools and daycares reopened, and from March 1^st^, secondary school students were allowed to have physical lessons again one day a week. On April 28^th^, non-essential shops and terraces reopened and on May 19^th^, it was again possible to visit leisure facilities, such as swimming pools and animal parks [18].

### Participants

In this cross-sectional study, two independent participant samples were included. The main study sample comprised children with a CSC who received treatment at an academic Dutch hospital. The control sample involved children from the Dutch general population.

### Children with CSC sample

Between December 3^rd^ 2020 and May 2^nd^ 2021 (hard lockdown including curfew), parents (of children aged 0–18 years) and children (aged 8–18 years) receiving long-term care at four academic Dutch hospitals (Emma Children’s Hospital, Amsterdam UMC; Sophia Children’s Hospital, Erasmus MC; Beatrix Children’s Hospital, UMC Groningen; Wilhelmina Children’s Hospital, UMC Utrecht) were invited to complete the COVID-19 child check questionnaire at home. This questionnaire was administered once for the current study, as part of the Patient Reported Outcome Measures (PROMs). PROMs are included in the standard care through the KLIK PROM portal (www.hetklikt.nu), which is an online portal to systematically monitor outcomes in children with various chronic diseases and their parents over time [19]. Parents and children of 8 years and older are asked to complete PROMs about health-related quality of life and psychosocial functioning prior to the outpatient consultation with the pediatrician or other healthcare professional. Answers on the PROMs are converted into a KLIK ePROfile and discussed during the consultation. KLIK is implemented in daily clinical practice since 2011 in > 30 Dutch hospitals for many different patient groups.

Healthcare professionals were asked to add the COVID-19 child check questionnaire to the already administered PROMs of their patients and to discuss the answers during the outpatient visit. For this study, we only used data of children and parents who gave online informed consent for use of their KLIK data for scientific purposes (83%).

### General population sample

Between November 6^th^ and 30^th^ 2020 (partial lockdown), research agency ‘Panel Inzicht’ invited parents with children aged 8–18 years from existing panels representative of the Dutch general population to complete the COVID-19 child check questionnaire. This procedure was part of other studies [8, 17]; we merely used the participants as a control group. The parents asked their children to complete the child-reported questions. The questionnaires were filled out on the research website of the KLIK portal. Data collection continued until a representative sample (within 2.5% variation on age and gender) of about 1,000 children was attained. The general population sample (8–18 years) included 1,214 children, with a mean age of 13.8 years and 48% boys.

### COVID-19 child check questionnaire

To detect the consequences of the COVID-19 pandemic for children and families at an early stage, a group of experts (pediatricians and psychologists, including KJ, KD and BdJvK) developed the COVID-19 child check questionnaire(Additional file 1), which was based on the CoRonavIruS health Impact Survey (CRISIS) [20]. The COVID-19 child check is intended as a tool for healthcare professionals to facilitate the conversation with children and parents about the impact of the COVID-19 pandemic they are experiencing.

Parents were asked to complete 5 questions about themselves and their family and 5 regarding their child. Children 8 years and older completed 4 questions about themselves. The questions regarding the parents themselves and the family concerned perceived stress (10-point Likert, from 1 (no stress) to 10 (extreme stress)) and change in family interaction, parenting perception, support, and financial situation (3 closed-ended responses and 1 open text option) since the start of the COVID-19 pandemic. Parents and children completed the same questions regarding the child’s perceived stress (10-point Likert, from 1 (no stress) to 10 (extreme stress)), coping with COVID-19 measures, and changes in time spent with friends and physical activity (3 closed-ended responses and 1 open text option) since the start of the COVID-19 pandemic. Parents completed an additional question about change in eating behavior of the child (closed-ended responses with open text option). Parent-reported support and financial situation were compressed into two response categories to meet the assumption of chi square tests that the expected value of cells should be 5 or greater in at least 80% of cells. That is, less support from others (such as family and friends) was combined with less support from care providers and the subdivision in being able or unable to make ends meet was combined into a group with deteriorated financial situation.

### Statistical analyses

Characteristics of children from both samples were described in means and percentages. For this study, the perceived stress item responses and the closed-ended responses of the COVID-19 child check questionnaire were analyzed. Child-reported outcomes of the two samples (8–18 years) were compared with t-tests and chi square tests, along with the parent-reported outcomes concerning the child (8–18 years). The parent-reported outcomes regarding themselves and the family were described for the complete CSC sample (0–18 years). As children 8 years and older filled out the COVID-19 child check themselves, we used this age as a cut-off. T-tests and chi square tests were used to compare parents of young children (0–7 years) with parents of older children (8–18 years) from the CSC sample and to compare parents from the CSC sample with parents from the general population (all having children aged 8–18 years). Additional t-tests and chi square tests were used to further explore associations with deteriorated financial situation. The association between the child’s perceived stress and parental perceived stress was examined with the Pearson’s correlation coefficient in both samples (8–18 years). SPSS software (IBM SPSS Statistics for Windows, Version 25.0. Armonk, NY: IBM Corp.) was used for all statistical analyses.

## Results

### Sample characteristics

The total CSC sample (0–18 years) included 326 children: mean age 10.9 years and 49% boys (Table 1). Participating children were recruited from academic hospitals in the North-Western part of the Netherlands from a variety of pediatric patient groups. Hematology (20%), rheumatology (18%) and congenital anomalies (12%) were the most frequent chronic diseases. Parents from the CSC sample had a mean age of 42.1 years and 78% were mothers. As for the sample of children aged 8–18 years in the CSC sample (n = 229), it comprised more girls than the general population sample aged 8–18 years (56% vs 48%, Χ^2^(1) = 4.39, *p* = 0.036), the mean age was not statistically different (13.6 y (SD 3.1) vs 13.8 y (SD 3.1), t(1135) = -0.62, p = 0.53).

**Table 1:** Characteristics of children and their parents (CSC sample, 0–18 years)

**Child characteristics**^a^	**Outcome**
Age, mean (SD)	10.9 (5.1)
Boys, %	49
Patient group, %	
Hematology	20
Rheumatology	18
Congenital anomalies	12
Gastroenterology	11
Endocrinology	6
Marfan syndrome	6
Dermatology	6
Other^b^	21
**Parent characteristics**	
Age, mean (SD)	42.1 (8.4)
Mothers/female guardian, %	78

^*a*^*N* = *326*

^*b*^*Including muscle diseases, viral infections, menstrual disorders, kidney transplantation, cystic fibrosis and ophthalmology*

### Impact of the COVID-19 measures on the children

**Fig. 1:**
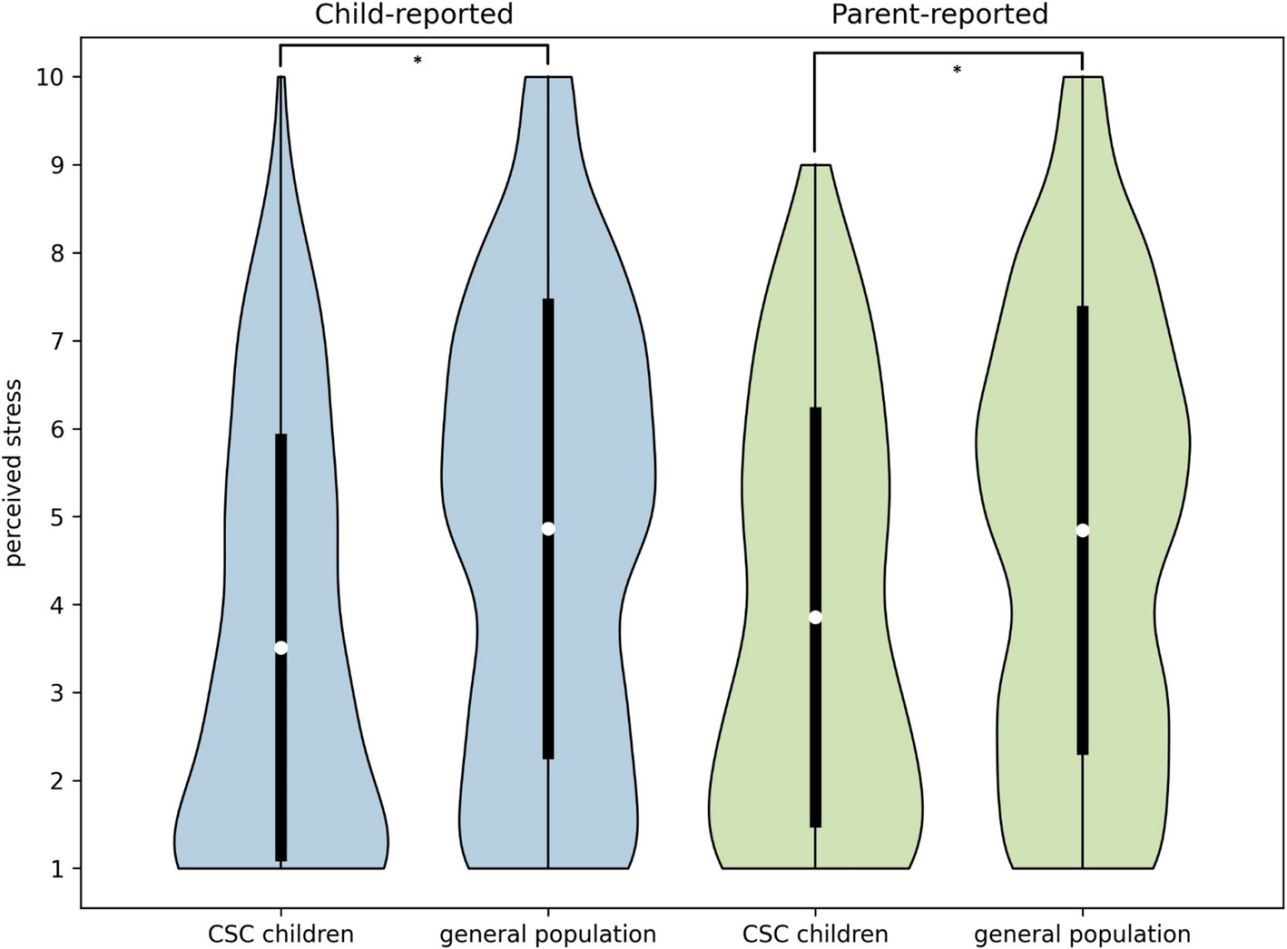
Distribution of child-reported and parent-reported perceived stress in Dutch children during COVID-19. *The white dots demonstrate the mean and the black bars the associated standard deviations*

Children (8–18 years) with CSC reported significantly lower stress levels (3.5 (SD 2.4) vs 4.9 (SD 2.6), t(1338) = -7.06, p < 0.001; stress scale: 1 (no stress) to 10 (extreme stress)) (Fig. 1), less social interaction with friends (59% vs 45%, Χ^2^(2) = 13.38, *p* = 0.001), and being less physically active (47% vs 30%, Χ^2^(2) = 25.46, *p* < 0.001) than the general population children (Table 2). Parents of the children with CSC (8–18 years) also reported less stress (3.9 (SD 2.3) vs 4.8 (SD2.5), t(1474) = -5.14, *p* < 0.001; stress scale: 1 (no stress) to 10 (extreme stress)), less social interaction with friends (55% vs 42%, Χ^2^(2) = 15.24, *p* < 0.001), and less physical activity (48% vs 26%, Χ^2^(2) = 38.01, *p* < 0.001) in their children compared with parents from the general population sample. More than 80% of the parents in both groups reported an unchanged eating behavior in their child, no difference between groups was found (Χ^2^(2) = 0.55, *p* = 0.76). Coping was not statistically different between the groups (child-reported Χ^2^(2) = 0.57, *p* = 0.75, parent-reported Χ^2^(2) = 2.58, *p* = 0.28), with about 60% of the children reacting neutrally towards the COVID-19 measures.

**Table 2:** Impact of the Dutch measures against COVID-19 on children aged 8–18 years

	**Child-reported**					**Parent-reported**				
	**CSC children**		**General population**			**CSC children**		**General population**		
	***N***	***Outcome***	***N***	***Outcome***	***p-value***	***N***	***Outcome***	***N***	***Outcome***	***p-value***
Perceived stress^a^, mean (SD)	*207*	3.5 (2.4)	*1,133*	4.9 (2.6)	** < 0.001**	*189*	3.9 (2.3)	*1,287*	4.8 (2.5)	** < 0.001**
Coping, %	*174*		*1,075*		0.75	*173*		*1,240*		0.28
Positive reaction		8		9			19		24	
Unchanged		64		62			62		57	
Negative reaction		28		29			18		18	
Social interaction with friends, %	*194*		*1,114*		**0.001**	*182*		*1,274*		** < 0.001**
See and speak to friends more often		4		7			2		8	
Unchanged		38		48			42		50	
See and speak to friends less often		59		45			55		42	
Physical activity, %	*202*		*1,122*		** < 0.001**	*183*		*1,277*		** < 0.001**
More physically active		6		13			5		13	
Unchanged		47		58			48		61	
Less physically active		47		30			48		26	
Eating behaviour, %						*175*		*1,277*		0.76
Healthier							10		12	
Unchanged							83		81	
Less healthy							7		8	

*Outcome values are means with standard deviations or percentages*

^*a*^*Assessed on a 10-point Likert scale (1 (no stress) to 10 (extreme stress))*

### Impact of the COVID-19 measures on the parents

Parents of children aged 0–18 years in the CSC sample reported a mean stress score of 4.1 (SD 2.2) (stress scale: 1 (no stress) to 10 (extreme stress)) for themselves (Table 3). The majority of these parents experienced no change in family interaction (80%), parenting perception (72%), support from others and care providers (85%), and financial situation (88%).

**Table 3:** Impact of the Dutch measures against COVID-19 on parents

	**CSC children** **0–18 y**		**CSC children** **0–7 y**		**CSC children** **8–18 y**		***p-value CSC 0–7 y vs 8–18 y***	**General population** **8–18 y**		***p-value CSC 8–18 y vs general population 8–18 y***
	***N***	***Outcome***	***N***	***Outcome***	***N***	***Outcome***		***N***	***Outcome***	
Perceived stress^a^, mean (SD)	*286*	4.1 (2.2)	*97*	4.0 (2.2)	*189*	4.1 (2.2)	0.86	*1,287*	5.1 (2.5)	** < 0.001**
Family interaction, %	*272*		*91*		*181*		0.82	*1,273*		0.11
More positive	*32*	12		12		12			15	
Unchanged	*217*	80		78		81			73	
More negative	*23*	8		10		8			11	
Parenting perception, %	*268*		*89*		*179*		0.56	*1,266*		**0.04**
Less difficult	*11*	4		2		5			11	
Unchanged	*194*	72		74		72			67	
More difficult	*63*	24		24		23			22	
Support, %	*267*		*93*		*174*		**0.049**	*1,270*		** < 0.001**
Unchanged	*228*	85		80		89			76	
Less support from others and care providers	*39*	15		20		11			24	
Financial situation, %	*276*		*94*		*182*		**0.02**	*1,260*		** < 0.001**
Unchanged	*244*	88		82		92			71	
Deteriorated, able or unable to make ends meet	*32*	12		18		8			29	

*Outcome values are means with standard deviations or percentages*

^*a*^*Assessed on a 10-point Likert scale (1 (no stress) to 10 (extreme stress))*

When splitting the CSC sample by age, parents of children with CSC aged 0–7 years did not differ in stress score with parents of children with CSC aged 8–18 years (4.0 (SD 2.2) vs 4.1 (SD 2.2), t(284) = -0.18, p = 0.86; stress scale: 1 (no stress) to 10 (extreme stress)), nor in parenting perception (Χ^2^(2) = 1.18, *p* = 0.56). Parents of children with CSC aged 0–7 years experienced less support (20% vs 11%, Χ^2^(1) = 3.88, *p* = 0.049) and more financial deterioration (18% vs 8%, Χ^2^(1) = 5.86, *p* = 0.02) than parents of children with CSC aged 8–18 years.

**Fig. 2:**
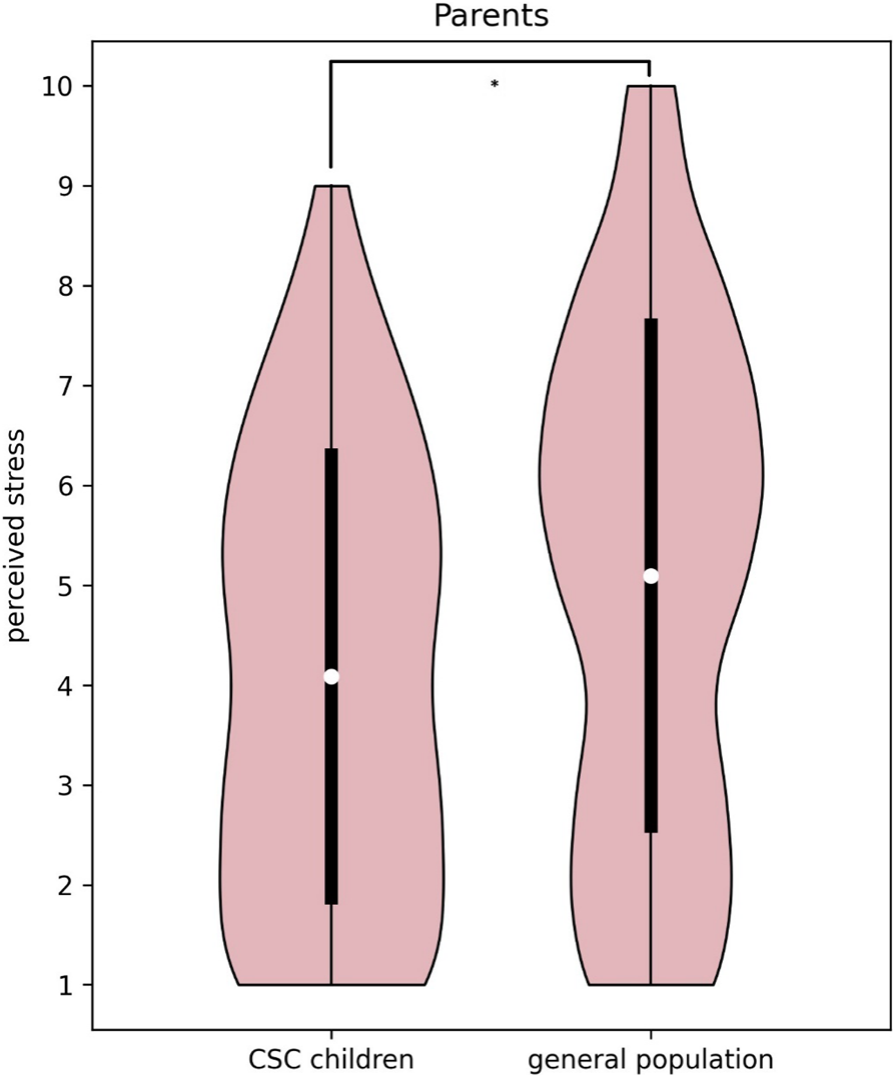
Distribution of perceived stress in Dutch parents during COVID-19. *The white dots demonstrate the mean and the black bars the associated standard deviations*

Parents of CSC children aged 8–18 years reported significantly less stress than parents of children aged 8–18 years in the general population (4.1 (SD 2.2) vs 5.1 (SD 2.5), t(1474) = -5.23, *p* < 0.001; stress scale: 1 (no stress) to 10 (extreme stress)) (Fig. 2). Parents from the general population more often indicated parenting as less difficult (11% vs 5%, Χ^2^(2) = 6.43, *p* = 0.04), received less support from others (24% vs 11%, Χ^2^(1) = 14.04, *p* < 0.001), and encountered more financial deterioration (29% vs 8%, Χ^2^(1) = 34.78, *p* < 0.001) than parents of CSC children. Additional analyses showed that a deteriorated financial situation among parents in the general population was associated with more parental stress (t(1258) = -6.32), worse family interaction (Χ^2^(2) = 36.06), worse parenting perception (Χ^2^(2) = 105.50), and less received support (Χ^2^(1) = 141.60), (all *p* < 0.001). These associations were not found in parents of CSC children (aged 8–18 years, nor in ages 0–18 and 0–7 years).

In the general population, the association between the perceived stress reported by the child and the perceived stress reported by the parents themselves (*r* = 0.64, *p* < 0.001) was stronger compared with the child-parent stress association in the CSC sample (*r* = 0.37, *p* < 0.001) (z-observed = -4.39).

## Discussion

Our study aimed to describe the impact of the Dutch COVID-19 measures on children with CSC and their parents and to compare them with a control group of children and parents from the general population. The impact of COVID-19 measures on perceived stress and physical and social daily life activities of children with CSC and their parents differed from those in the general population. Children in the CSC sample engaged less in physical activity and had less social interaction with their friends during the COVID-19 measures compared with children from the general population sample. On the other hand, both children and their parents in the CSC sample reported less stress compared with those in the general population sample. There was a difference depending on the age of the child within the CSC sample, parents of children aged 0–7 years experienced less support and more financial deterioration than parents of children aged 8–18 years. Surprisingly, this deteriorated financial situation was not associated with perceived stress or daily life impact whereas in the general population these associations were significant in parents of children 8–18 years.

We expect that the different impact of COVID-19 measures on perceived stress and daily life in children with CSC and their parents compared with those in the general population could not be explained by the different inclusion periods in the COVID regulation timeline in the Netherlands. The Dutch COVID-19 restrictions were more tightened during the inclusion of the CSC sample with temporarily school closures and a curfew. Therefore, it could be presumed that the impact on children from the general population and the differences between the CSC and general population samples might even be underestimated in our results.

We found lower perceived stress in children with CSC and their parents compared with those in the general population. This finding is in line with a Dutch study that demonstrated less mental health problems among children with pre-existing somatic conditions compared with children from the general population and compared with children having pre-existing psychiatric conditions (all aged 8–18 years) during the Dutch COVID-19 lockdown in April–May 2020 [17]. In contrast, a study in the US found lower stress levels among parents with healthy children than among parents of children with chronic somatic or mental conditions [15]. This difference may be explained by the fact that we explicitly addressed somatic conditions, potential differences in healthcare access, differences in the assessment of stress, and by assessing different populations of children. Our study used a 10-point Likert scale to explore stress during the COVID-19 pandemic among parents, whereas the US study used the Perceived Stress Scale (PSS). According to the PSS, the stress levels among the parent groups in the US study (healthy children, children with chronic conditions) were both denoted as ‘moderate’, which could indicate that despite the differences in perceived stress, the clinical relevance of these differences, however, might be limited. Disease-specific studies also showed varying results regarding psychological impact. Children with chronic lung diseases and their parents in Turkey had more anxiety than healthy controls during COVID-19 [13]. This finding is comprehensible with COVID-19 generally being known as a lung disease. In children with cystic fibrosis, however, COVID-19 had no effect on anxiety levels, but anxiety in their mothers was raised [14]. Dutch children with cancer and their parents reported lower stress during COVID-19 than before COVID-19 [21]. One could argue that children with chronic diseases are used to a certain amount of stress and might have developed coping strategies, for example related to school absence or being distant from friends, which allow them to cope effectively with any additional stress due to COVID-19 regulations [22–24]. Moreover, the COVID-19 measures might have reduced prior everyday demands that normally caused stress. It could also be argued that stress in children with CSC is lower because COVID-19 measures impose the avoidance of certain activities and they do not have to decide for themselves whether they participate or that their parents may be more shielding [25]. We recommend analyses considering disease type and severity in future research to examine this hypotheses. Besides, qualitative research may provide more insight into underlying reasons of given stress levels and help to further develop the COVID-19 child check questionnaire.

As to financial changes since COVID-19, both parents of younger (0–7 years) children with CSC and parents from the general population experienced more deterioration during COVID-19 measures than parents of 8–18 year old CSC children. This could be explained by adaptation practices. Depending on the type of disease, financial and time caregiving burden in children with CSC are generally higher than for healthy children [26, 27]. Consequently, families of older children (8–18 years) have adapted to this situation over time, for instance with adjusted career choices, financial aid and support, and altered expenditure patterns [27, 28]. The same is likely regarding support, as parents of (older) children with CSC may already have built up a sustainable network on which they rely [28]. The absence of an association between financial deterioration and parental stress or other family impact in the CSC sample could also be attributed to earlier adaptation. Since financial deterioration was associated with more perceived stress and negative family impact in the general population sample, one could argue that these families have not yet adapted and therefore faced more family life disruptions due to COVID-19.

As to physical and social daily activities, it is known that children with chronic conditions exercise less and have less social interaction with friends compared with healthy children [5]. Our findings demonstrated that these behaviors in children with CSC were also more negatively influenced by the COVID-19 measures, with a striking 59% of children that saw and spoke to friends less often and 47% that was less physically active than before COVID-19. Although physical activity remained the same in the majority of children from the general population, more than a quarter stated to have been less physically active during COVID-19 measures. This is in line with other literature that found a reduction of physical activity among children during COVID-19 restrictions, along with increased screen time behavior [29–32]. Our findings urge for attention to physical activity and social interaction with friends for all children both during and after COVID-19 measures. Although children with CSC reported less stress, their less engagement in physical activity and social interaction with friends are worrisome. Given the disadvantages children with CSC already had before the COVID-19 pandemic in these areas, and the fact that participation in sports and non-digital social interactions also benefits their wellbeing and development [33, 34], interventions targeting physical and social activity on the long term is of great importance and beneficial specifically in this population of children.

The weak association between child and parental stress during COVID-19 measures in the CSC sample might be another sign of adaptation. Stress in children and parents from the general population was associated more strongly. This observed difference in stress association between children and parents could be attributed to specific characteristics of both samples or different coping mechanisms.

### Strengths and limitations

The strengths of this study are a broad spectrum of child and family outcomes and the inclusion of a large control group from the general population. The CSC sample was relatively small which impaired sub-analyses among different patient groups, besides there was no information available on disease severity. Due to the cross-sectional design of the study, results on a possible temporal relation between COVID-19 measures and the outcomes were hampered and causal conclusions could not be drawn. Lastly, the (psychometric) validity and reliability of the COVID-19 child check questionnaire have not been investigated yet. However, since the questionnaire primarily serves as a signaling tool and does not measure one specific construct, validation may be difficult.

## Conclusions

This study provides evidence of positive as well as negative consequences of the Dutch COVID-19 measures in children with CSC and their parents. While children with CSC experienced less stress, they had less social interaction with friends and engaged less in physical activity during Dutch COVID-19 measures than children in the general population. As to clinical implications, it is recommended to monitor whether they resume these activities in the long run. Children and parents from the general population reported more stress, more often had a deteriorated financial situation, and experienced less support than the children with CSC and their parents. As long as COVID-19 prohibits return to normal daily life, questionnaires such as the COVID-19 child check could assist healthcare professionals in discussing problems. By revealing the collateral damage of COVID-19 measures among children and their families, the COVID-19 child check might also guide policy when considering new measures or supporting children, for example in reducing stress or promoting physical activity.

## Supplementary Information

**Additional file 1.** COVID-19 child check questionnaire (.doc).

## Data Availability

The datasets used and analyzed during the current study are available from the corresponding author on reasonable request.

## Abbreviations

COVID-19: Coronavirus disease 2019
CSC: Chronic somatic conditions
