# Supplementary material for: Perceived stress, family impact, and changes in physical and social daily life activities of children with chronic somatic conditions during the COVID-19 pandemic
Source: BMC Public Health. 2022 Jun 3;22:1106. doi: 10.1186/s12889-022-13544-8 (PMC9163527; doi:10.1186/s12889-022-13544-8)
Supplement: Supplementary file 1 — Additional file 1. COVID-19 child check questionnaire (.doc). [file 12889_2022_13544_MOESM1_ESM.docx]

**Additional file 1**

**COVID-19 child check questionnaire**

**Parent part**

The following questions are about changes in your family and with your child during the Corona period, from March 2020 up to now.

Questions about you and your family

1. How much stress do you experience in the current Corona period?

1 2 3 4 5 6 7 8 9 10

No stress Extreme stress

1. Do your family members interact differently during the Corona period?

a.) My family members interact more positively.

b.) My family members interact the same as always.

c.) My family members interact more negatively, for example, there is more arguing or irritation.

d.) Other: ….

1. How do you experience parenting during the Corona period?

a.) I do not experience any change in parenting.

b.) I find parenting less difficult than before the Corona period.

c.) I find parenting more difficult than before the Corona period.

d.) Other: ….

1. Has the support you receive from others, such as family, friends or care providers, changed during the Corona period?

a.) I get as much support from others as always.

b.) I get less support from others, such as family and friends.

c.) I get less support from care providers.

d.) Other: ….

1. Has the financial situation of your family changed during the Corona period?

a.) Nothing has changed in our financial situation.

b.) Our financial situation has deteriorated, but we are able to make ends meet.

c.) Our financial situation has deteriorated; we have trouble making ends meet.

d.) Other: ….

Questions about your child

1. How much stress does your child experience in the current Corona period?

1 2 3 4 5 6 7 8 9 10

No stress Extreme stress

1. How does your child react to the measures during the Corona period?

a.) My child reacts positively to the measures.

b.) My child reacts neutrally to the measures.

c.) My child reacts angry, sad or frustrated to the measures.

d.) Other: ….

1. Has anything changed in your child’s eating behavior during the Corona period?

a.) My child has been eating healthier.

b.) Nothing has changed in my child’s eating behavior.

c.) My child has been eating less healthy.

d.) Other: ….

1. Has anything changed in the amount of physical activity of your child during the Corona period, for example, in playing outside, sports and cycling?

a.) My child has been more physically active.

b.) Nothing changed in how much my child has been physically active.

b.) My child has been less physically active.

d.) Other: ….

1. Has Corona changed anything in how often your child sees or speaks to friends?

a.) My child sees or speaks to friends more often.

b.) Nothing has changed in how often my child sees or speaks to friends.

c.) My child sees or speaks to friends less often.

d.) Other: ….

**Child part**

1. How much stress do you experience due to Corona?

1 2 3 4 5 6 7 8 9 10

No stress Extreme stress

1. How do you feel because of the Corona measures?

a.) I experience positive feelings because of the measures.

b.) The measures do not affect how I feel.

c.) I experience negative feelings, such as sadness or anger, because of the measures.

d.) Other: ….

1. Has Corona changed anything in how often you see or speak to your friends?

a.) I see or speak to my friends more often.

b.) Nothing has changed in how often I see or speak to my friends.

c.) I see or speak to my friends less often.

d.) Other: ….

1. Has anything changed in the amount of physical activity during this Corona period, for example in playing outside and sports?

a.) I have been more physically active.

b.) Nothing has changed in how much I am physically active.

c.) I have been less physically active.

d.) Other: ….
